# MicroRNA-31 Function as a Suppressor Was Regulated by Epigenetic Mechanisms in Gastric Cancer

**DOI:** 10.1155/2017/5348490

**Published:** 2017-12-03

**Authors:** Jun Wei, Zijian Wang, Zhixiang Wang, Yong Yang, Changlai Fu, Jianqing Zhu, Danbin Jiang

**Affiliations:** Department of Gastroenterology, Yancheng Affiliated Hospital of Southeast University, Yancheng 224000, China

## Abstract

Gastric cancer is one of the most lethal malignancies worldwide. The aberrant expression of microRNA-31 (miR-31) has been reported in gastric cancer; however, its regulation mechanisms are still unclear. Here, we confirmed that miR-31 expression was significantly decreased in gastric cancer tissue and cell lines. Ectopic expression of miR-31 potentially suppresses proliferation and induced early apoptosis in gastric cancer cells. Furthermore, miR-31 expression was regulated as a result of epigenetic mechanisms. The downregulation of miR-31 was associated with promoter DNA methylation status in gastric cancer and cell lines. Moreover, we found that* HDAC2* was the direct target of miR-31 by binding to 3′-UTR from the results of luciferase reporter assays, qRT-PCR, and western blotting. HDAC2 played an activation role in tumor growth, whose expression is upregulated and inversely associated with miR-31 levels. All the results suggested that miR-31 function as a crucial tumor suppressor was regulated by epigenetic mechanisms in gastric cancer. We found an epigenetic pathway loop, DNA methylation-miRNA expression-target gene-tumor progression in gastric cancer, and also provided implications for molecular diagnosis and therapeutics of gastric malignancies by detecting miR-31 as a potential target.

## 1. Introduction

Gastric cancer is a malignant cancer and associated with a high mortality worldwide, especially in developing countries [1]. In China, there are approximately 700,000 new cases and 500,000 deaths every year [2]. Due to lack of definite symptoms during early disease stages, most patients are diagnosed at advanced stages [3]. Currently, because no curative treatments are available for gastric cancer therapy, the five-year survival rate for advanced stage gastric cancer patients is less than 20% [4].

Cancer is a complex disease caused by the genetic factor and environment. Accumulating evidences have suggested that various genetic alterations contributing to the development of gastric cancer, the loss of several tumor suppressors, and aberrant regulation of cellular growth signaling pathway are associated with tumorigenesis [5].

Aberrant regulation of noncoding RNAs, especially miRNA, has been proposed to be associated with many kinds of cancer. miRNAs are short noncoding RNAs with 21–25 nucleotides which target mRNAs for degradation or translational repression by direct binding to the 3′-UTR of targeting gene [6]. The involvement of miRNAs in cancer pathogenesis is well established that aberrant expression of miRNAs can play critical roles in tumorigenesis by inducing oncogenes, inhibiting tumor suppressor genes, or disrupting signaling pathways [7]. miRNAs also can be as oncogenes or tumor suppressor genes depending on the cellular function of their targets [8].

microRNA-31 (miR-31) is one of the most frequently altered miRNAs in human cancers, whose expression and functions were extensively studied and well defined in many kinds of cancer [9]. It is reported that miR-31 was downregulated in ovarian and prostate cancer and glioma; meanwhile miR-31 was upregulated in colorectal, lung, and pancreatic cancer [10]. In gastric cancer, miR-31 was of low expression [11, 12]; however, the roles of miR-31 are still unidentified, and the regulation of miR-31 was also unclear.

Epigenetic mechanisms, such as noncoding RNA and DNA methylation, play critical roles during cancer development [13, 14]. Promoter cytosine methylation at specific gene loci has a profound impact on gene expression, genome stability, and chromatin structure [15]. Hypomethylation of the gene promoter region is associated with activation, whereas hypermethylation is a feature of gene silence. Furthermore, DNA methylation also regulated miRNA expression through methylated specific promoter locus [16, 17]. It has reported that miRNA expression was associated with DNA methylation in prostate cancer [18, 19]. However, whether the expression of miR-31 was associated with promoter methylation alteration in gastric cancer remained unclear. Meanwhile, little is known about the association between miRNA promotor methylation, expression, and gastric cancer pathogenesis.

In this study, we found that overexpression of miR-31 potentially suppresses gastric cancer cell proliferation and induced apoptosis. The downexpression of miR-31 was associated with promoter DNA methylation in gastric cancer. Moreover, HDAC2 was the direct target of miR-31 by binding to 3′-UTR, whose expression is upregulated and inversely associated with miR-31 levels. All the results suggested that miR-31 functions as a tumor suppressor through the regulation of epigenetic mechanisms in gastric cancer.

## 2. Material and Methods

### 2.1. Clinical Samples

Gastric cancer biopsy specimens from 52 GC patients (29 males and 23 females) were collected from Yancheng Affiliated Hospital of Southeast University from May 2014 to May 2015. The informed consent was received from all patients before anticipation. The research protocol was according to the Declaration of Helsinki and approved by the Ethics Committee of Yancheng Affiliated Hospital of Southeast University. The diagnoses of gastric cancer were confirmed by pathology, and the patients had not received any radiotherapy or chemotherapy before surgery. GC tissue and adjacent normal tissue were collected during surgery using digestive endoscopy and stored at −70°C until use.

### 2.2. Cell Culture

Gastric cancer cell lines BGC-823, SGC-9701, and AGS and normal gastric cell lines GES-1 were purchased from Cell Bank of Chinese Academy of Sciences, an agent of American Type Culture Collection in China [20]. Cells were cultured in DMEM medium supplemented with 10% fetal bovine serum (Gibco, US), 100 U/ml of penicillin, and 100 mg/ml streptomycin (Gibco, US) in incubator (Thermo Fisher, US) with a 5% CO_2_ at 37°C.

### 2.3. Cell Proliferation Assay

The cell proliferation was determined with the CCK-8 kit (Dojindo, Japan). 3,000 cells were seeded into 96-well plates for 24 hours and then treated with NC.miR (miRNA control), miR-31 mimics, or miR-31 AS (antisense). After 24, 48, 72, and 96 hours of treatment, 100 *μ*l cultural supernatant was collected to another 96-well plate and then 10 *μ*l CCK-8 solution was added for incubating at 37°C for 4 hours. The absorbance at 450 nm wavelengths was measured using spectrophotometry (BioTek, US).

### 2.4. Cell Apoptosis Assay

Cells were seeded in 6-well plates and treated with NC.miR, miR-31 mimics, or miR-31 AS. At indicated time points (0, 24, 48, and 96 hours), 2 × 10^5^ cells were washed twice with 1 mL cold PBS and fixed with 1 mL 4% w/v paraformaldehyde on ice for 60 minutes. The fixed cells were washed once with PBS and 10 *μ*l Annexin V-FITC solution was added (Beyotime, China) at room temperature in the dark for 10 minutes. Then, the cell pellets were collected, and 10 *μ*l propidium iodine solution was added (Beyotime, China). After being mixed gently, the cell suspensions were detected with flow cytometry (BD, US) within 15 minutes.

### 2.5. RNA Extraction and Real-Time PCR

Total RNA was extracted from cells with Trizol reagent (Takara, Japan) and identified by gel electrophoresis and Nanodrop (Thermo Fisher, US); then the genome DNA was digested by DNase I (Takara, Japan). cDNA was synched using reverse transcriptase (Takara, Japan). Quantitative real-time PCR assays were performed in Applied Biosystems 7500 Systems (ABI, US) using SYBR Green PCR Mix (Takara, Japan). The following primer was used: miR-31 (5′-TAATACTGCCTGGTAATGATGA-3′ and 5′-GTCGTATCCAGTGCAGGGTCCGAGGTATTCGCACTGGATACGACAGCTAT-3′), U6 (5′-GCGCGTCGTGAAGCGTTC-3′, 5′-GTGCAGGGTCCGA GGT-3′), HADC2 (5′-GGTGCTGGAAAAGGCAAATA-3′, 5′-ACGGATTGTGT AGCCACCTC-3′), and *β*-actin (5′-ATGATATCGCCGCGCTCG-3′, 5′-CGCTCGGTGA GGATCTTCA-3′). The reaction conditions were 95°C 5 minutes, then 95°C 10 seconds, 60°C 10 seconds, and 72°C 15 seconds for 40 cycles; melt curve was detected at the end. The mRNA relative expression levels were quantified by 2^(−ΔΔCt)^ method and each value then related to the corresponding internal control as well as the U6 or GAPDH.

### 2.6. Western Blotting

The total protein was lysed from gastric cancer cells using RIPA solution (Sangon, China) and quantitated with BCA kit (Beyotime, China). 20 *μ*g cell total protein was heated at 105°C for 5 minutes and then separated by SDS-PAGE. The separated gels were transferred onto a PVDF membrane (Millipore, US) using a Bio-Rad machine (US). The transferred PVDF membrane was incubated with 5% nonfat milk in PBS containing 0.05% Tween-20 (PBST) for 1 hour and then incubated with primary antibody at 25°C for 4 hours. After being washed four times with PBST, the membrane was incubated with horseradish peroxidase (HRP) conjugated secondary antibody for another 2 hours. Detection was performed by using a chemiluminescent ECL detection kit (Thermo Fisher, US). The primary antibodies of *β*-actin, HDAC2, caspase 3, and the HRP conjugated second antibodies were purchased from Santa Cruz (US).

### 2.7. Invasion Assay of GC Cell Lines

Transwell plates (BD, US) were used for gastric cancer cell invasion assay. The bottom chamber has complete media and the upper chamber has serum-free media. Matrigel (BD, US) was added to the DMEM medium for detecting invading cells. After transfection of NC.miR or miR-31 mimics, gastric cancer cells were appropriately seeded into the cell culture. After incubation at 37°C for 20 hours, the invaded cells were fixed and stained using Giemsa. The images of cells were photographed with microscope (Olympus, Japan) at ×100 magnification and the cell number was counted in three random fields of view. The results were shown using column graph with statistics.

### 2.8. Luciferase Reporter Assays

The plasmids psi-CHECK2-HDAC2-3′-UTR-wt and psi-CHECK2-HDAC2-3′-UTR-mut were luciferase reporter vector which contained wild type or mutant of the miR-31-binding site on HDAC2 gene 3′-UTR [9, 21]. These two plasmids were constructed following the above reference. For luciferase reporter assays, the gastric cancer cells were plated at a density of 1.0 × 10^5^ cells per well in 96-well plates and grown to 70% confluence. The psi-CHECK2-HDAC2-3′-UTR-wt or psi-CHECK2-HDAC2-3′-UTR-mut was transiently transfected into cells together with the* Renilla* reporter plasmid pRL (Promega, US) using Lipofectamine 3000 Reagent (Life, US) according to the instruction. Firefly and* Renilla* luciferase activities were measured at 48 hours after transfection using Dual-Luciferase Reporter Assay System (Promega, US). Firefly luciferase activity was normalized to* Renilla* luciferase activity as relative luciferase activity. The relative activity was expressed as the mean ± s.d. of triplicate or quadruplicate wells of a representative experiment performed three times.

### 2.9. Sodium Bisulfate Sequencing

Genomic DNA was extracted from gastric cancer tissue or cell lines using QIAamp DNA Mini kit (Qiagen, German). Sodium bisulfate sequencing was performed using EZ DNA Methylation Kit (Enyo, US) according to the manufacturer's instructions [22]. Briefly, 2 *μ*g DNA was treated according to the instructions. The modified DNA was amplified with primers (5′-AGGAAGAGAGTTTTTTTTAAGAAGGGAAAGTTTAG-3′ and 5′-CAGTAATACGACTCACTATAGGGAGAAGGCTCAAATAAACTAAA AAAACCTTAATCCC-3′) specific for the miR-31 promoter region. The PCR products were cloned into pMD18T vector (Takara, Japan) and at least 10 clones were sequenced for each experiment to analyze the DNA methylation status. The DNA methylation level was analyzed with sequencing data.

### 2.10. Statistical Analysis

All the results are expressed as mean ± standard deviation and the differences between data groups were evaluated by Student's *t*-test. *p* values less than 0.05 were considered statistically significant (^*∗*^*p* < 0.05, ^*∗∗*^*p* < 0.01, and ^*∗∗∗*^*p* < 0.01).

## 3. Results and Discussion

### 3.1. miR-31 Was Aberrantly Downregulated in Gastric Cancer and Cell Lines

**Figure 1:**
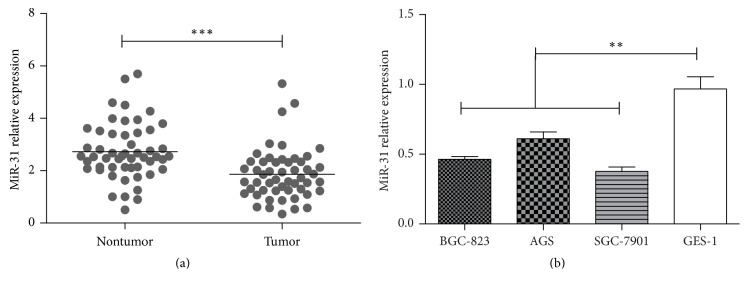
*miR-31 was aberrantly downregulated in gastric cancer and cell lines.* The relative expression of miR-31 was detected by qRT-PCR analysis in 52 randomly selected gastric cancer tissues paired with adjacent noncancerous gastric tissues (a) and gastric cancer cell lines BGC-823, SGC-7901, and AGS and gastric normal cell line GES-1 (b). The expression of miR-31 was normalized to U6 snRNA (^*∗∗*^*p* < 0.01, ^*∗∗∗*^*p* < 0.001).

miR-31 is one of the most frequently altered miRNAs in human cancers. It is reported that miR-31 downregulation has been detected in ovarian and prostate cancer and glioma; meanwhile miR-31 was upregulated in colorectal, lung, and pancreatic cancer [23]. The miR-31 expression in GC was a paradox; there are some papers stating that miR-31 is highly expressed in GC cell lines, and some reported that miR-31 is significantly downregulated. To furtherly investigate the expression of miR-31 in gastric cancer, 52 randomly selected GC tissues paired with adjacent noncancerous gastric tissues were detected by quantitative real-time PCR (qRT-PCR). The results have shown that the expression of miR-31 was significantly downregulated compared to corresponding nontumor tissue (Figure 1(a)). This result confirmed that miR-31 expression was suppressed in gastric cancer tissue.

Gastric cancer tissues exhibited significantly downregulation of miR-31 in patients (Figure 1(a)). Furthermore, endogenous expression of miR-31 was investigated in different gastric cell lines, including BGC-823, SGC-7901, AGS, and GES-1. It is shown that the gastric cancer cell lines (BGC-823, SGC-7901, and AGS) exhibited relatively low miR-31 expression levels compared to normal gastric cell lines GES-1 (Figure 1(b)). BGC-823 and SGC-7901 cells have shown significantly lower expression of miR-31, and AGS cells exhibited relatively high expression of miR-31 in three GC cells. These results implied that the expression of miR-31 is inhibited in gastric cancer and its low expression may be associated with biological process of tumorigenesis. The expression alteration in gastric cancer cell lines might be associated with differentiation status. AGS cell lines exhibited high level of cell differentiation, SGC-7901 moderate level of cell differentiation, and BGC-823 low level of cell differentiation.

### 3.2. miR-31 Suppressed Gastric Cancer Cell Proliferation

**Figure 2:**
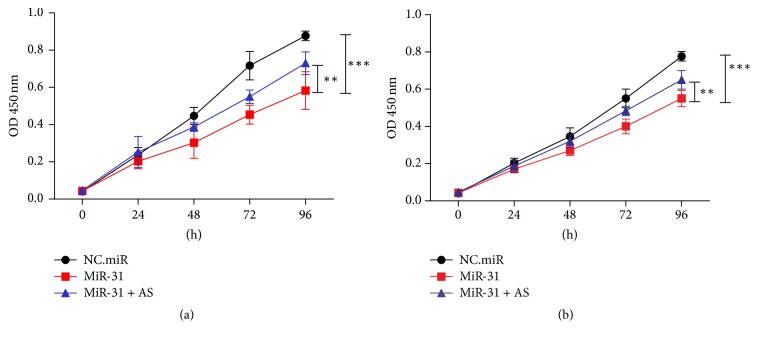
*miR-31 suppressed gastric cancer cell proliferation.* NC.miR (negative control), miR-31 mimics (ectopic expression), or miR-31 + AS (antisense miR-31) were transfected into BGC-823 (a) and SGC-7901 (b) cell lines. The cell growth rate was determined by measuring CCK-8 absorbance at A_450_ at every 24 hours (^*∗∗*^*p* < 0.01, ^*∗∗∗*^*p* < 0.001).

It is well known that miRNAs play roles in all processes of cancer, including apoptosis and proliferation. To investigate functions of miR-31 in gastric cancer cell proliferation, we utilized ectopic expression of miR-31 and measured cell growth of two gastric cancer cell lines BGC-823 and SGC-7901 using CCK-8 method. The results have shown that overexpression of miR-31 resulted in reduced growth rates of BGC-823 and SGC-7901 cell lines, whereas cotransfection with miR-31-AS (an antisense inhibitor of miR-31) significantly blocked this antigrowth effect (Figure 2). This effect could be caused by the disruption of cell growth regulation on miR-31 targeting gene, such as cell cycle arrest or apoptosis. Similar results were reported in other research groups, including gastric cancer, hepatocarcinogenesis, and prostate cancer [9, 11, 19], indicating that a connection between miR-31 and cancer cell proliferation was a general mechanism.

### 3.3. miR-31 Induced Early Apoptosis in Gastric Cancer Cell

**Figure 3:**
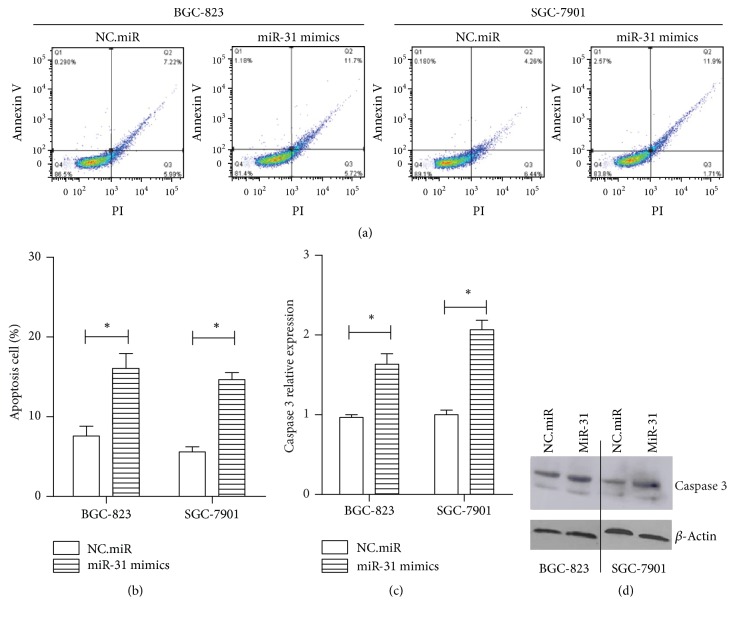
*miR-31 induced early apoptosis in gastric cancer cell.* (a, b) 48 hours after transfection of NC.miR or miR-31 mimics, apoptosis assay was assessed to determine apoptosis rate of BGC-823 and SGC-7901 cells. (c) The total RNA was extracted from transfected cells after 24 hours, and the mRNA level of caspase 3 was measured by real-time PCR. *β*-Actin was an internal control. (d) The total protein was extracted from transfected cells with RIPA solution after 48 hours, and caspase 3 protein was detected by western blotting. *β*-Actin was an internal control (^*∗*^*p* < 0.05).

Following the above results of cell proliferation, to investigate biological functions of miR-31 in gastric cancer cells apoptosis, BGC-823 and SGC-7901 cells were treated with NC.miR or miR-31 mimics for 24 hours and then detected apoptosis rate using double dyeing of Annexin V and PI; the apoptosis cells were counted using flow cytometry. It was found that ectopic expression of miR-31 induced early apoptosis in these two gastric cancer cells (Figures 3(a) and 3(b)). The early apoptosis rate was increased in BGC-823 (12% versus 7%) and SGC-7901 (12% versus 6%) cells after overexpression of miR-31. Interestingly, the total apoptosis rate (early and late) was similar in all groups. Moreover, caspase 3, an apoptosis related marker protein, was detected by real-time RT-PCR and western blotting. After being transfected with miR-31 mimics, the caspase 3 RNA level increased about 1-fold, but the protein level had no significant change in both cells (Figures 3(c) and 3(d)), in keeping with the total apoptosis level. In previous studies, serval researchers reported that the miR-31 had no significant effect on cell apoptosis in gastric cancer or hepatocarcinogenesis [9, 11], but some reporters have shown that overexpression of miR-31 induced apoptosis in SGC-7901 cells [12].

### 3.4. miR-31 Suppressed Invasion of Gastric Cancer Cell

**Figure 4:**
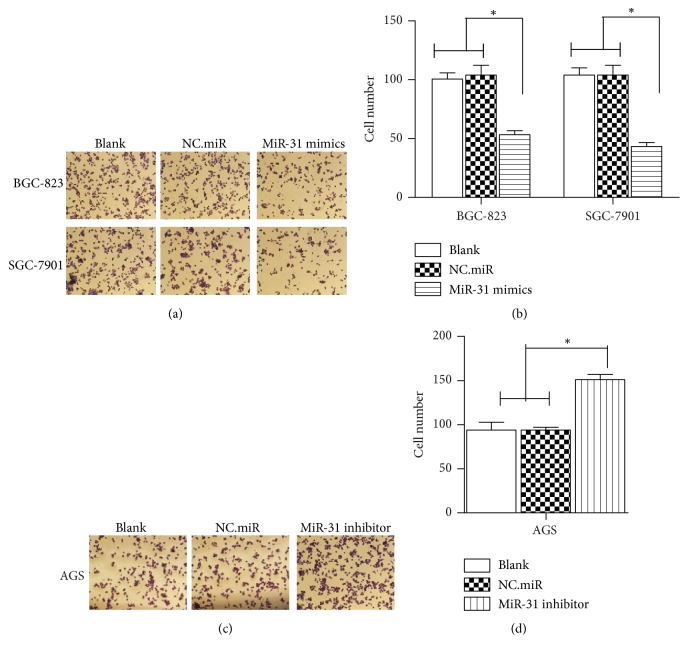
*miR-31 suppressed invasion of gastric cancer cells.* (a) NC.miR or miR-31 mimics were transfected into BGC-823 and SGC-7901 cells. (c) NC.miR or miR-miR-31 inhibitor was transfected into AGS cells. After 24 hours, invasion assay was performed with transwell plates. Images were taken with microscope. (b, d) The cell number of invasion cells were counted in 5 randomly selected fields (^*∗*^*p* < 0.05).

To further evaluate the role of miR31 on the progression and metastasis of gastric cancer, the cell invasion was performed. As shown in the results, transfection with miR-31 mimics significantly restricted the invasion rates in the BGC-823 and SGC-7901 cells compared to the blank and NC.miR groups (Figures 4(a) and 4(b)). In contrast, transfection with miR-31 inhibitor significantly increased the invasion rates in the AGS cell lines compared to the blank and NC.miR groups (Figures 4(c) and 4(d)). These data were similar with the previous reporters and suggest that miR-31 effectively inhibited tumor cell invasion of gastric cancer cells in vitro.

### 3.5. miR-31 Was Regulated by Epigenetic Mechanisms in Gastric Cancer

It is well known that epigenetic alterations can result in dysregulation of miRNA expression [24]. To delineate the mechanism behind the downregulation of miR-31 in gastric cancer, we investigated whether epigenetic regulation might account for the miR-31 expression.

**Figure 5:**
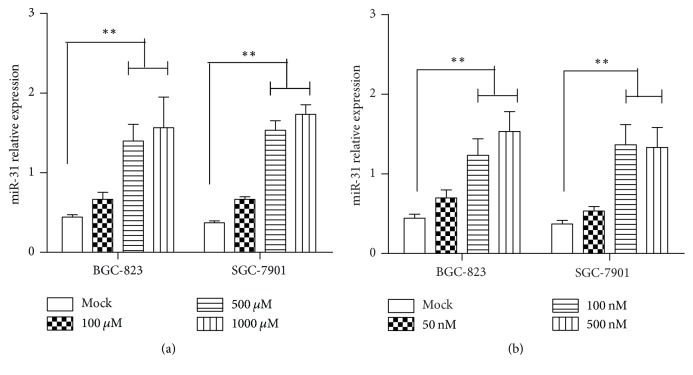
*miR-31 expression was regulated by HDACi and DNMTi.* The expression of miR-31 was measured by qRT-PCR in BGC-823 and SGC-7901 cell lines, which were treated with TSA for 24 h (a) and DAC for 24 h (b). The relative expression was normalized with U6 (^*∗∗*^*p* < 0.01).

Firstly, the miR-31 expression was measured in gastric cancer cells treated with HDACi (histone deacetylase inhibitor) or DNMTi (DNA methyltransferase inhibitor). It is shown that TSA, an HDACi, increased miR-31 expression with dose-dependent manner both in BGC-823 and in SGC-7901 cell lines (Figure 5(a)). Meanwhile, the expression of miR-31 is upregulated by DAC, a DNMTi (Figure 5(b)), in dose-dependent manner. Generally, epigenetic regulation was an important role in cancer biology; drugs of HDACi and DNMTi were approved for therapeutics of cancer. Moreover, it had reported that HDACi can differentially regulate expression of miRNAs in cancer cells [25]. miR-31 was a target of HDACi in breast cancer cells [26]. Thus, our finding of miR-31 upregulation by epigenetic mechanisms in gastric cancer was logical and implied that miR-31 expression in gastric cancer might be regulated by epigenetics mechanisms.

**Figure 6:**
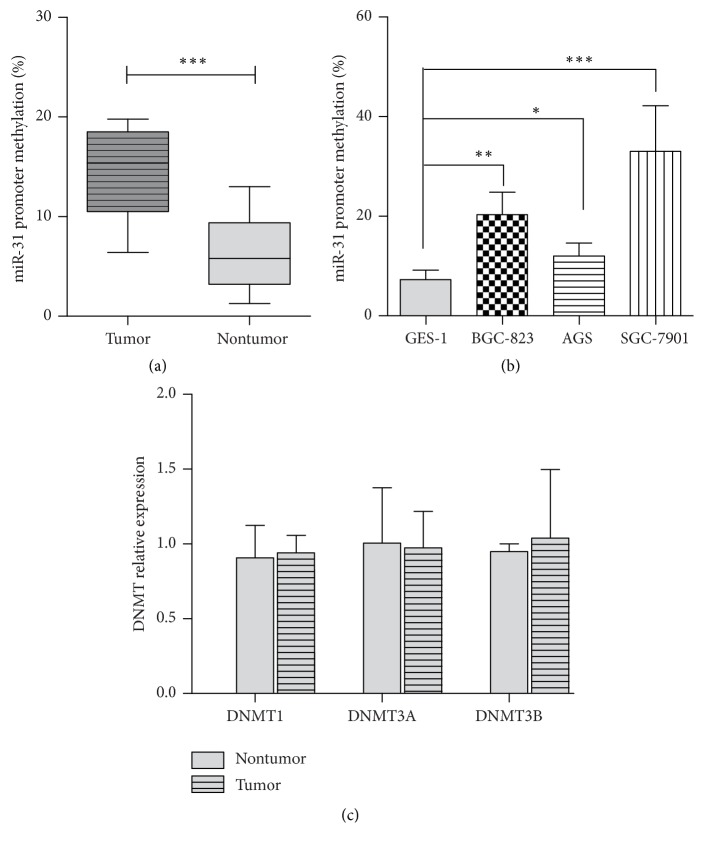
*miR-31 promoter was hypermethylated in gastric cancer.* The genome DNA was extracted from gastric cancer tissue (a) or cell lines (b); the methylation level of miR-31 promoter was detected using bisulfite sequencing PCR. (c) The total RNA was extracted from gastric cancer tissue; the expression of DNMT1, 3A, and 3B was measured by qRT-PCR (^*∗*^*p* < 0.05, ^*∗∗*^*p* < 0.01, and ^*∗∗∗*^*p* < 0.001).

Promoter DNA methylation was associated with gene and miRNA expression [17, 27]. To investigate the underling roles in gastric cancer, the methylation level of miR-31 promoter was measured. We found that the miR-31 promoter showed cancer-specific hypermethylation status. The gastric cancer tissue displayed significantly higher methylation levels of miR-31 promoter than matched gastric tissues (15.5% versus 6.1%) (Figure 6(a)). Meanwhile, the promoter methylation level was also examined in gastric cancer cell line. The human gastric epithelial cell line GES-1, which had high expression of miR-31, has shown lower DNA methylation on the promoter region. In contrast, gastric cancer cells such as BGC-823, SGC-7901, and AGS with low miR-31 expression had concurrent high DNA methylation levels (Figure 6(b)). Taken together, promoter DNA methylation levels were inversely correlated with miR-31 expression, suggesting that promoter hypermethylation accounts for miR-31 downregulation in the gastric cancer.

To furtherly delineate the reason of miR-31 promotor methylation alteration, DNA methyltransferase level was measured. As shown in Figure 6(c), DNMT expression was not significantly different between tumor and nontumor tissue (similar to gastric cancer cell lines, data not shown), implying that the miR-31 promoter methylation alternation was not caused by DNMT expression in gastric cancer tissue or cell lines.

### 3.6. miR-31 Regulated HDAC2 in Gastric Cancer

**Figure 7:**
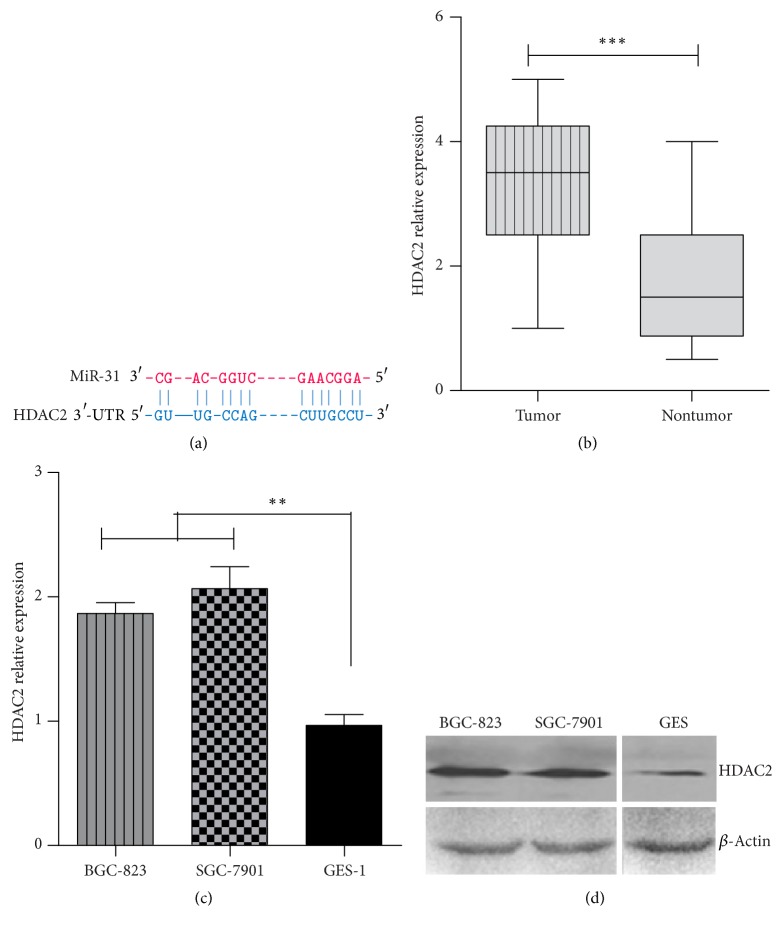
*HDAC2 was upregulated in gastric cancer.* (a) The target sites of miR-31 on 3′-UTR of HDAC2 are shown as a schematic representation. The qRT-PCR analysis HDAC2 expression in 52 paired gastric cancer tissues (b) and cell lines (c). (d) The protein levels of HDAC2 in BGC-823, SGC-7901, and GES-1 cell lines were detected by western blot analysis (^*∗∗*^*p* < 0.01, ^*∗∗∗*^*p* < 0.001).

The downregulation of miR-31 will cause genes aberrant expression in gastric cancer. To identify miR-31 target genes in gastric cancer, firstly we used several prediction programs, including miRWALK, Targetscan, and miRtarbase. From these databases, about 400 genes were predicted to be targeted by miR-31. Secondly, we scanned potential target genes of miR-31 by reviewing publications. Considering epigenetic mechanisms, we pay attention to the* HDAC2* (histone deacetylase 2) gene which had a miR-31 binding site in 3′-UTR (Figure 7(a)). Subsequently, the expression of HDAC2 was detected in gastric cancer tissue and cell lines. As an exception, HDAC2 was significantly overexpressed in gastric tumor group compared to nontumor group (Figure 7(b)). Meanwhile, the gastric cancer cell lines (BGC-823, SGC-7901) also exhibited relatively high expression levels compared to normal gastric cell lines GES-1 (Figures 7(c) and 7(d)).

We predicted and confirmed that HDAC2 might be a target gene of miR-31 in gastric cancer. Previously, increased expression of histone deacetylase 2 was found in human gastric cancer [28]. From results of two large cohorts of gastric cancer patients (GSE24375 and GSE13196), HDAC2 gene expression was significantly upregulated in gastric cancer [29].

**Figure 8:**
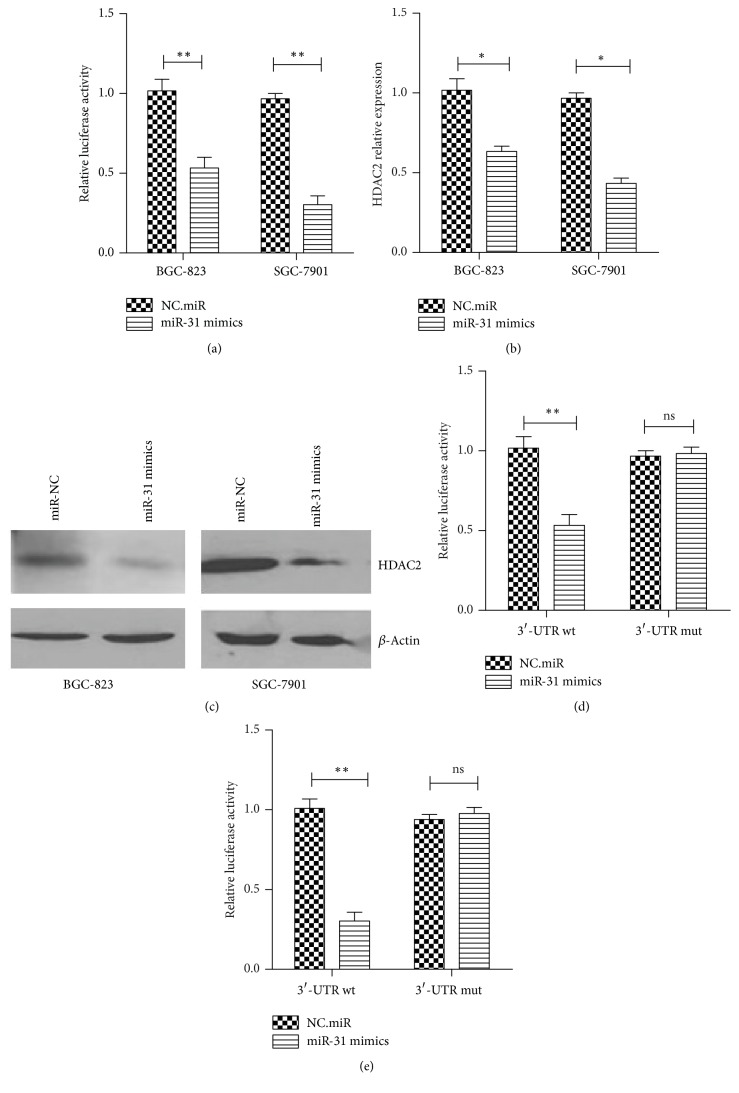
*miR-31 regulated HDAC2 expression in gastric cancer.* (a) Wild type psi-CHECK2-HDAC2-3′-UTR-wt plasmid was cotransfected with miR-31 mimics into BGC-823 and SGC-7901 cells. Relative luciferase activity was detected using dual luciferase system. The mRNA relative expression (b) and protein level (c) of HDAC2 were detected in BGC-823 and SGC-7901 cells which was transfected with miR-31 mimics or NC.miR. Wild type or mutant HDAC2 3′-UTR plasmid was cotransfected with miR-31 mimics or NC.miR in BGC-823 (d) and SGC-7901 cells (e). Relative luciferase activity was detected. (^*∗*^*p* < 0.05, ^*∗∗*^*p* < 0.01, ns, no significance).

To investigate whether HDAC2 was directly regulated by miR-31 in gastric cancer, the 3′-UTR sequence of HDAC2 was cloned into a luciferase reporter vector to construct psi-CHECK2-HDAC2-3′-UTR-wt plasmid. We found that the relative luciferase activity of HDAC2 3′-UTR was inhibited by miR-31 in BGC-823 and SGC-7901 cell lines (Figure 8(a)). Moreover, the mRNA relative expression and protein level of HDAC2 were also suppressed in those two cell lines (Figures 8(b) and 8(c)). These results suggested that HDAC2 may be targeted by miR-31 in gastric cancer. Next, to verify that miR-31 specifically binds to 3′-UTR of HDAC2, mutant luciferase vector psi-CHECK2-HDAC2-3′-UTR-mut of the 3′-UTR of HDAC2 was constructed and then transfected into BGC-8232 and SGC-7901 cell lines. The results have shown that miR-31 was able to suppress wild type reporter vector activity, whereas mutant plasmids showed no changes in both cells, indicating that miR-31 selectively regulates both HDAC2 expressions in gastric cancer cells (Figures 8(d) and 8(e)). These results demonstrate that miR-31 directly regulates HDAC2 expression in gastric cancer cells.

**Figure 9:**
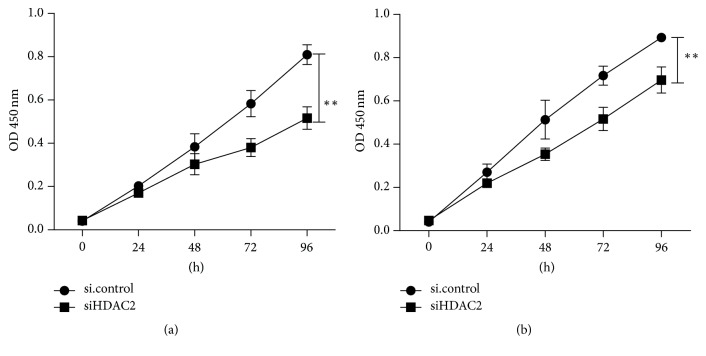
*Downregulation of HDAC2 suppressed gastric cancer proliferation.* siHDAC2 was transfected into BGC-823 (a) and SGC-7901 (b) cell lines. The cell growth rate was determined by measuring CCK-8 absorbance at A_450_ at every 24 hours (^*∗∗*^*p* < 0.01).

As a target gene of miR-31 in gastric cancer, we confirmed the HDAC2 roles in gastric cancer cell proliferation using siHDAC2 method. As shown in Figure 9, downregulation of HDAC2 resulted in reduced growth rates of BGC-823 and SGC-7901 cell lines. As an important histone deacetylase, the roles of HDAC2 were studied in many cancers. It is reported that inactivation of HDAC2 can restore p16^*INK4a*^ activity and have antitumor effects on gastric cancer [29]. It is well known that HDAC2 expression was associated with poor prognosis and possibly contributes to cancer progression [30, 31].

## 4. Conclusion

In this study, we found an epigenetic pathway loop, DNA methylation-miRNA expression-target gene-tumor progression in gastric cancer. miR-31 expression was significantly decreased in gastric cancer tissue and cell lines. Artificial ectopic expression of miR-31 potentially suppressed gastric cancer cell proliferation and induced early apoptosis. Furthermore, miR-31 expression was regulated by epigenetic mechanisms. The downregulation of miR-31 was associated with promoter methylation status in gastric cancer and cell lines. Moreover, we identified that HDAC2 was the direct target of miR-31 by binding to 3′-UTR. Downregulation of HDAC2 suppressed gastric cancer proliferation, whose expression is upregulated and inversely associated with miR-31 levels. All the results suggested that miR-31 function as a crucial tumor suppressor was regulated by epigenetic mechanisms in gastric cancer and also provided implications for molecular diagnosis and therapeutics of gastric malignancies by detecting miR-31 as a potential target.
